# A Rare Association Between Osteomalacia, Phosphaturic Mesenchymal Tumor, and Ovarian Cancer: A Case Report and Literature Review

**DOI:** 10.1007/s00223-024-01231-2

**Published:** 2024-05-28

**Authors:** Marcodomenico Mazza, Gaetano Paride Arcidiacono, Ilda Hoxhaj, Virginia Padoan, Giulia Tasca, Marta Burei, Stefania Sella, Paolo Simioni, Sandro Giannini, Simone Mocellin

**Affiliations:** 1 Soft-Tissue, Peritoneum and Melanoma Surgical Oncology Unit, Veneto Institute of Oncology IOV-IRCCS, Padua, Italy; 2 Department of Medicine, Clinica Medica 1, University of Padova, Via Giustiniani 2, Padua, Italy; 3 Department of Surgical, Oncological and Gastroenterological Sciences (DISCOG), University of Padova, Padua, Italy; 4 Oncology 2 Unit, Veneto Institute of Oncology IOV-IRCCS, Padua, Italy; 5 Nuclear Medicine Unit, Veneto Institute of Oncology IOV-IRCSS, Padua, Italy

**Keywords:** Tumor-induced osteomalacia, Ovarian cancer, FGF23, Phosphaturic mesenchymal tumor

## Abstract

Tumor-induced osteomalacia (TIO) is a rare paraneoplastic syndrome characterized by hypophosphatemia, bone mineralization disorders with increased risk of fragility fractures, muscle pain, and progressive weakness. TIO has been associated with increased production of the phosphaturic hormone Fibroblast Growth Factor 23 (FGF23) usually by mesenchymal tumors of soft tissue or bone (Phosphaturic Mesenchymal Tumors—PMTs). In rare cases TIO may be observed in association with other malignancies. We report the case of a 66-year-old woman with an occasional diagnosis of both a PMT and an ovarian cancer during the evaluation of TIO. We also systematically review the literature to discover possible correlations between osteomalacia, FGF23 production, and ovarian cancer. Four studies were eligible for the analysis. Two case reports described an association between TIO development and ovarian cancer, whereas the two case-control studies hypothesized a possible correlation between FGF/FGF receptor axis and cancer development. Although it does not provide conclusive evidence regarding the association between TIO and ovarian cancer, this case report highlights the possibility that in the diagnostic workup of suspected TIO, both FGF23-secreting tumors distinct from PMT and tumors unrelated to the clinical presentation of TIO could be identified. This information is important for guiding successful tumor staging and determining the necessity for surgical intervention and/or eventual adjuvant therapy.

## Introduction

Tumor-induced osteomalacia (TIO), also known as “oncogenic osteomalacia,” is a rare paraneoplastic syndrome characterized by renal phosphate wasting, resulting in moderate or severe hypophosphatemia, bone mineralization disorders with increased risk of fragility fractures, muscle pain, and progressive weakness [1]. First described by McCance [2] and Prader [3], TIO has been associated with increased production of the phosphaturic hormone Fibroblast Growth Factor 23 (FGF23), usually by mesenchymal tumors of soft tissue or bone known as “Phosphaturic Mesenchymal Tumors” (PMTs) [4]. FGF23, normally secreted by osteocytes and osteoblasts, acts on proximal renal tubular epithelial cells to regulate phosphorus homeostasis. Excess FGF23 leads to the internalization of sodium-phosphate cotransporters, reducing renal phosphate reabsorption and causing hyperphosphaturia. Additionally, FGF23 inhibits intestinal phosphate absorption by reducing the conversion of 25-OH-vitamin D into its active form, 1,25-OH2-vitamin D (or calcitriol) [4]. PMTs are quite rare, and the vast majority reported are clinically benign. However, a minority of cases show malignant behavior, with distant metastases and multifocal disease (about 10% of cases) [5, 6]. In rare cases FGF23-mediated TIO has been reported in patients with other types of tumors, including prostate cancer [7, 8], breast cancer [9], colon adenocarcinoma [10], and renal clear cell carcinoma [11]. In these cases, typically the cancer diagnosis is already known at the time of detecting hypophosphatemia and osteomalacia. However, the primary neoplasm may be incidentally detected during the diagnostic workup for suspected TIO [11]. We report the case of a 66-year-old woman in whom both a PMT and an ovarian granulosa cell tumor were identified during the diagnostic evaluation of a TIO. Because of the rarity of the two neoplasms and the not usual association between ovarian cancer and TIO, we reviewed current literature according to the Preferred Reporting Items for Systematic Reviews and Meta-Analyses (PRISMA) guideline [12]. The aim of the study is to summarize the evidence of a possible correlation between oncogenic osteomalacia and ovarian cancer and to report our experience.

## Case Presentation

**Fig. 1:**
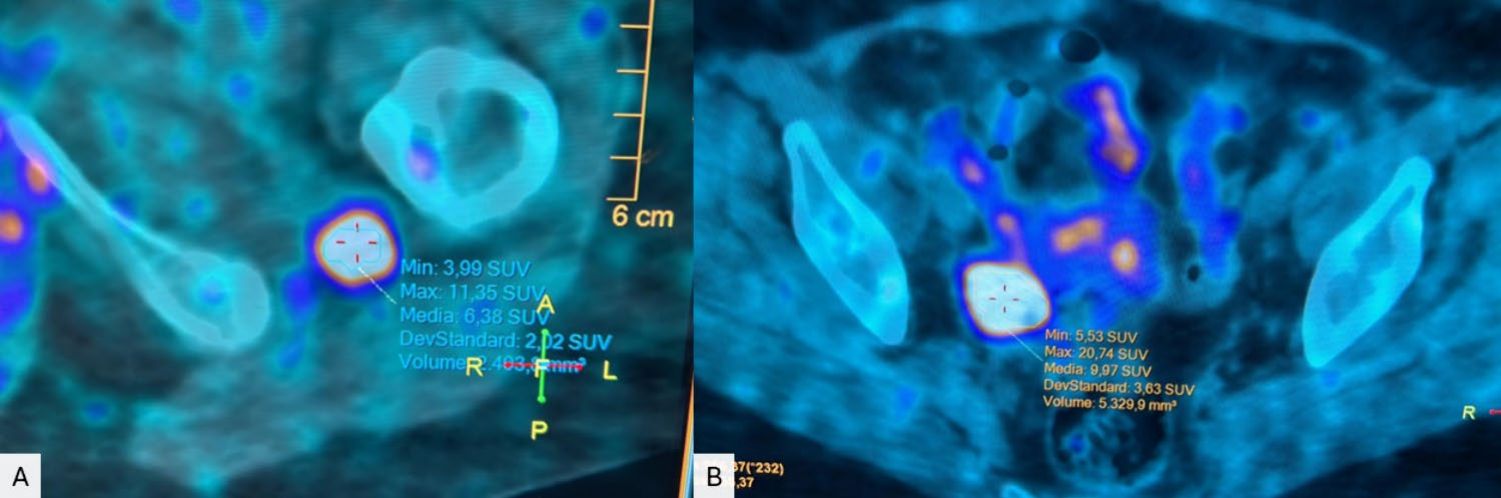
^68^Ga-DOTATOC PET/CT showing tracer uptake in a nodule in the left quadratus femoris muscle (**A**) and in a nodule in the right para-uterine region (**B**). ^68^Ga-DOTATOC PET/CT: ^68^Ga-DOTA-D-Phe^1^-Tyr^3^-octreotide positron emission tomography/computed tomography

After a 3-year history of generalized weakness, widespread muscular pain, and multiple bone fractures (ribs, metatarsus, ankle, bilateral ischiopubic branches), a 66-year-old Caucasian woman underwent a diagnostic work-up for bone fragility in 2022. Her remote personal and familial medical history was not relevant, and she was only taking cholecalciferol supplements (2500 IU/day). A bone densitometry (Hologic, Waltham, MA) revealed a lumbar spine T-score of − 1.1, total hip T-score of − 1.6, and femoral neck T-score of − 1.9. As shown in Table 1, laboratory tests revealed severe hypophosphatemia (0.42 mmol/L), associated with increased total (ALP, 265 U/L) and bone-specific alkaline phosphatase (bALP 150 µg/L). Additionally, there was an increase in other bone turnover markers (C-terminal telopeptide of type I collagen or CTX, and N-terminal propeptide of type I procollagen, or P1NP) and serum parathyroid hormone (PTH) levels. Serum and urinary levels of calcium, creatinine, and 25-OH-vitamin D were within the normal range. Suspecting a hypophosphatemic osteomalacia, we measured the maximum rate of tubular phosphate reabsorption to the glomerular filtration rate (TmP/GFR), which was below the age- and gender-specific reference range [13], suggesting renal phosphate wasting. Moreover, the level of 1,25-OH_2_-vitamin D was inappropriately low in the context of hypophosphatemia, and both intact and C-terminal FGF23 levels were elevated (Table 1). Medical treatment with sodium phosphate (2500 mg/day) and calcitriol (0.5 µg/day) was started, resulting in only a mild increase in serum phosphate (0.61 mmol/L). Considering the absence of family history and the late age of onset, genetic forms of hypophosphatemic osteomalacia were excluded and a ^68^Ga-DOTATOC (^68^Ga-DOTA-D-Phe^1^-Tyr^3^-octreotide) PET/TC (Positron Emission Tomography/Computed Tomography) was then performed. It showed increased uptake in a nodule located in the left quadratus femoris muscle (with a standardized uptake value [SUV] of 11.35) and in a nodule situated in the right para-uterine region (maximum SUV 20.74) (Fig. 1A, B). A subsequent contrast-enhanced computed tomography (CT) scan confirmed the presence of a hyperdense formation measuring 3 cm in the right para-uterine region and a hyperdense nodular formation measuring 16 mm in proximity to the left ischial tuberosity. After multidisciplinary discussion, the patient underwent surgery with removal of the pertrochanteric nodule and right video-laparoscopic adnexectomy en bloc with the nodule was also performed. The histopathologic examination of the pertrochanteric nodule showed spindle cells characterized by fibrous stroma with multifocal powdery calcifications. Immunohistochemistry was positive for FGFR1 and SATB2. The analysis confirmed the diagnosis of PMT. On the contrary, the right ovarian lesion resulted in an adult-type granulosa cell tumor (AGCT). To complete tumor staging, the patient underwent laparoscopic peritoneal biopsies, omentectomy, left adnexectomy, hysterectomy, and right pelvic lymphadenectomy, resulting negative for tumor localization (pTNM T1aN0M0, FIGO IA according to American Joint Commission on Cancer (AJCC) 8th edition [14]. No adjuvant treatment was considered due to the early-stage and the histology of the ovarian cancer. The course of the hospitalization was regular and without complications. After surgery, the patient’s symptomatology subsequently regressed with significant improvement in pain and energy levels. Phosphate supplements and calcitriol were progressively tapered until discontinuation. After four months, the patient underwent laboratory tests that documented initial biochemical recovery from osteomalacia. As shown in Table 1, serum phosphate and TmP/GFR values were within normal range, as well as the levels of intact and C-terminal FGF23. There was also a marked reduction in both total and bone alkaline phosphatase, considered markers of osteomalacia [15]. Six months later, as shown in Table 1, serum phosphate and TmP/GFR remained within normal range and there was a further decrease in the levels of total and bone alkaline phosphatase, as well as other markers of bone turnover.

**Table 1:** Laboratory exams at initial assessment and during follow-up

Laboratory exam	Initial assessment^a^	One month before surgery^b^	Four months after surgery^a^	Ten months after surgery^a^	Laboratory reference range
Serum creatinine (µmol/L)	52	–	61	62	45–84
Serum calcium (mmol/L)	2.28	–	2.47	2.38	2.10–2.55
Serum phosphate (mmol/L)	**0.42**	**0.61**	1.21	1.09	0.87–1.45
24-h urine calcium (mmol/24 h)	3.68	–	3.17	3.48	2.50–7.50
TmP/GFR (mmol/L)	**0.43**	–	1.21	1.00	0.72–1.29
25-OH-vitamin D (nmol/L)	89	–	104	103	75–250
1,25-OH_2_-vitamin D (pmol/L)	81.1	–	188.0	183.0	47.8–190.3
PTH (ng/L)	**54.0**	–	25.0	23.8	6.5–36.8
ALP (U/L)	**264**	–	**132**	**115**	33–98
bALP (µg/L)	**150.0**	–	**38.8**	**27.9**	4.7–27.1
CTX (pg/mL)	715	–	–	526	142–1351
P1NP (µg/L)	**165**	–	–	105	28–128
iFGF23 (pg/mL)	**108.0**	–	39.7	–	23.2–95.4
cFGF23 (pmol/L)	**2.3**	–	0.5	–	0.0–0.8

– Not available, *ALP* alkaline phosphatase, *bALP* bone alkaline phosphatase, *CTX* C-terminal telopeptide of type I collagen, *cFGF23* C-terminal fibroblast growth factor 23, *iFGF23* intact fibroblast growth factor 23, *PTH* parathyroid hormone, *TmP/GFR* ratio of the maximum rate of tubular phosphate reabsorption to the glomerular filtration rate, *P1NP* N-terminal propeptide of type I procollagen

Values outside reference ranges are highlighted in bold

^a^Not taking phosphate salts nor calcitriol

^b^Taking sodium phosphate (2500 mg/day) and calcitriol (0.5 µg/day)

## Literature Review

### Methods

In order to identify eligible studies, we systematically searched MEDLINE/PubMed, EMBASE, and SCOPUS. The search strategy was performed without language restrictions through September 2023. In PubMed, the following search strategy was used: *((“Oncogenic osteomalacia” [Supplementary Concept]) OR “Fibroblast Growth Factor-23”[Mesh]) AND “Ovarian Neoplasms”[Mesh].* The search strategy was tailored to fit the other electronic sources. The lists from each source were joined and the duplicates were removed. Two investigators (MM, VP) separately evaluated the full-text of all records in order to remove those not fulfilling the inclusion criteria. Finally, the reference lists of included records were hand-searched to discover further studies of interest. Any disagreement was solved by consensus with a third investigator (IH). Case series, cohort studies, and case-control studies reporting information on TIO cases in ovarian cancer patients were eligible for inclusion. Two investigators (MM, VP) independently extracted relevant data from the included articles. For each article, study features (year of publication, study design, aim, and sample size), patient and clinical characteristics (age, phosphorus, and FGF-23 serum level), and outcome measures (reduction of FGF23 level) were collected. A third investigator (IH) checked the extracted data, and any inconsistency was solved by consensus.

### Results

**Fig. 2:**
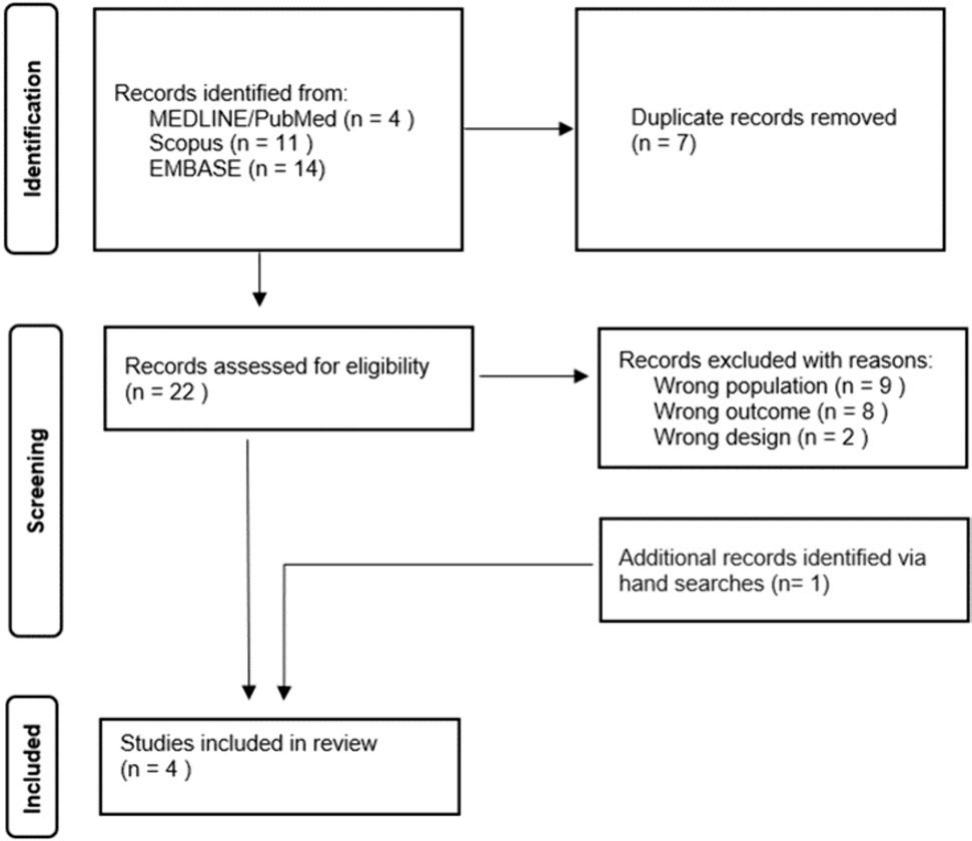
Flow-chart of selection process

The search identified 22 non-duplicated records. After exclusion of 19 records due to wrong design (*n* = 2), wrong outcomes (*n* = 9) or wrong population (*n* = 8), 3 records were included in the synthesis [16–18]. One more record was identified via hand-search [19]. The PRISMA flow-chart is shown in Fig. 2. Two of included studies were case–control conducted in the USA, one in 2005 [16] and the other one in 2014 [18], whereas two case-reports were published in Taiwan and India, in 2014 [17] and 2022 [19], respectively. Study characteristics of included studies are summarized in Table 2.

**Table 2:** Patients and laboratory features of included studies

First author	Year	Study design	Number of patients	Age at diagnosis, years: mean or median (range)	Symptoms	Serum phosphate level (normal range)	FGF23 concentrations (normal range)	Aim of the Study
Tebben et al. [16]	2005	Case–control	14 (benign)	60 (38–87)	–	3.2 ± 0.4 mg/dL (2.5–4.5 mg/dL)	30.5 ± 16 RU/mL (0–85 RU/mL)	To evaluate the correlation between serum/plasma FGF23 concentrations and ovarian neoplasm and to determine the association with disordered phosphate metabolism
			14 (early-stage)	57 (35–82)	–	3.2 ± 0.4 mg/dL (2.5–4.5 mg/dL)	16.2 ± 14 RU/mL (0–85 RU/mL)	
			13(advanced-stage)	69 (55–81)	–	3.6 ± 0.5 mg/dL (2.5–4.5 mg/dL)	247 ± 399 RU/mL (0–85 RU/mL)	
Lin et al. [17]	2014	Case report	1	57	Low back pain, weight loss and night sweats	1.6 mg/dL (2.7–4.5 mg/dL)	501.6 pg/mL → 44.141 pg/mL. (8.2–54.3 pg/mL)	To report a case of ovarian cancer-related hypophosphatemic osteomalacia
Meng et al. 18]	2014	Case-control	339	60.73 (–)	–	–	–	To investigate the correlation between FGF receptor genetic variants and risk of ovarian cancer or therapeutic response to chemotherapy or overall survival
Paul et al. [19]	2022	Case report	1	63	Osteoporosis, lower limb pain and proximal myopathy	0.6 mg/dL (2.5–4.6 mg/dL)	3200 RU/mL → 88 RU/mL (21–91 RU/mL)	To report the case of a TIO syndrome secondary to an ovarian teratoma

– Not available, *FGF* fibroblast growth factor, *FGF23* fibroblast growth factor 23, *TIO* tumor-induced osteomalacia

The case-report studies showed the features of TIO secondary to an epithelial ovarian carcinoma (Lin et al.) and cystic ovarian teratoma (Paul et al.). Lin et al. [17] reported the case of a woman with low back pain, weight loss, and night sweats six months prior to diagnosis. Hypophosphatemia, elevated serum alkaline phosphatase level, and low phosphorus tubular reabsorption rate were suggestive of TIO, but an MRI revealed multiple spinal metastases and stenosis in the lumbar spine. The diagnosis of stage IV ovarian carcinoma was considered given the elevated cancer antigen 125 level and CT findings of multiple tumors in the bone, lung, thyroid, liver, and a large tumor over the uterus and left adnexa. Neoadjuvant chemotherapy with carboplatin/paclitaxel was initiated, followed by debulking surgery, and the histopathological examination confirmed high-grade serous ovarian carcinoma. On the other hand, Paul et al. [19] described the case of a 63-year-old postmenopausal woman presenting characteristics of TIO secondary to an ovarian teratoma. The patient complained of aching pain predominantly in her lower limbs and worsening proximal myopathy started six months before diagnosis. Due to persistent hypophosphatemia with hyperphosphaturia, TIO was considered. She had a high level of FGF23, but a ^68^Ga-DOTATATE (^68^Ga -DOTA-0-Tyr^3^-octreotate) PET/CT did not reveal any DOTA-avid lesion. A laparoscopic bilateral salpingo-oophorectomy was performed, with total excision of the left adnexal lesion, which histological examination revealed a mature cystic ovarian teratoma. In both cases, normalization of serum FGF23 levels was observed following surgical removal of the lesions.

Case-control studies evaluated the association of FGF23 concentrations with ovarian cancer stage [16] and of various FGF single nucleotide polymorphisms (SNPs) with cancer development risk, survival, and therapeutic response [18].

Tebben et al. [16] measured serum or plasma FGF23 concentrations in 39 healthy controls and in 14 women with benign ovarian tumors, 14 with early-stage, and 13 with advanced-stage ovarian cancer. A significant positive correlation was found between serum FGF23 concentrations and stage of disease, but no patient with an elevated FGF23 concentration had hypophosphatemia. Moreover, FGF23 was detected in tissue staining in malignant ovarian cancer cells. Meng et al. [18] matched 339 ovarian cancer cases with 349 healthy controls and genotyped them for 183 SNPs from 24 FGF and FGF receptor (FGFR) genes. FGF1 SNP rs7727832 was associated with ovarian cancer, whereas ten SNPs were associated with a reduced risk. FGF23 rs7961824 was the most significantly associated with improved prognosis. FGF18 SNP rs3806929, FGF7 SNP rs9920722, FGF23 SNP rs12812339, and FGF5 SNP rs3733336 were significantly associated with a favorable treatment response.

### Critical Appraisal of the Quality of Included Studies

Two investigators (MM, VP) independently appraised the quality of the included studies according to the critical appraisal tool of the Joanna Briggs Institute (JBI) [20]. A third investigator (IH) checked the assessments, and any inconsistency was solved by consensus. Case reports clearly describe patients’ characteristics, medical history, symptoms and severity of the condition, diagnostic tests, treatment and post-intervention follow-up and summarized key lessons learned. In case–control studies, the groups were comparable and were matched properly; the exposure was measured in the same way in cases and control outcomes were assessed in a reliable way; whereas it was not clear whether it was controlled for confounding factors.

## Discussion

TIO is a rare paraneoplastic syndrome commonly associated with PMTs, a histotype that in 2013 was included in the “Classification of Tumors of Soft Tissue and bone” by the World Health Organization [21]. Most PMTs occur in middle-aged adults involving the extremities and acral sites, although very rare cases have been reported in infants and the elderly. Detecting PMTs can be challenging due to their small size (with a diameter ranging between 2 and 14 cm) and slow benign growth, sometimes the primary tumor remains unknown [22]. A recent global guidance for the diagnosis and management of TIO suggests using a systemic approach to tumor localization, which begins with functional imaging and is followed by anatomic imaging [22]. ^68^Ga-based or ^64^Cu-based PET/CT (^68^Ga/^64^Cu-DOTATATE, -DOTANOC, or -DOTATOC) are suggested as first-line functional imaging. Instead, magnetic resonance imaging and computed tomography, depending on the suspected location, are generally recommended as anatomic imaging due to their high resolution [1]. In some cases, venous sampling of FGF23 levels can be used to localize or confirm a suspected causal tumor or to distinguish between two possible lesions observed in functional imaging [23]. Surgical resection, with a complete excision, is the treatment of choice leading to the reduction of serum FGF23 levels and resolution of osteomalacia in most of the patients [24]. In patients with non-localizable, non-resectable, or recurrent tumors, medical treatment becomes crucial, as it aims to improve phosphate homeostasis and alleviate clinical symptoms. Traditionally, it includes both phosphate salts and active vitamin D analogs (calcitriol or alfacalcidol), but the emergence of burosumab, a monoclonal antibody targeting FGF23, represents a promising alternative for those unable to undergo surgery or unresponsive to conventional therapy [25]. In our case, the clinical suspicion of TIO led to the execution of a ^68^Ga-DOTATOC PET/CT scan, which documented two lesions with tracer hyperuptake, one in the left quadratus femoris muscle and the other in the right parauterine region. Given that 46.4% of PMTs are localized in the lower limbs [26], we hypothesized that TIO could be attributed to the lesion found in the left quadratus femoris muscle. Due to the rarity of phosphaturic tumors in the pelvic region (10.3%), we opted not to perform a venous sampling but to proceed directly with surgical excision of both lesions, suspecting that the one in the right parauterine region might be a neoplasm of different nature. Although it is recommended to obtain a histopathological diagnosis before surgery for a bulky abdominal mass [27], we avoided performing a CT-guided percutaneous biopsy due to the size and localization of the lesion. In the case report by Paul et al. [19], a core needle biopsy was not performed so the patient underwent bilateral salpingo-oophorectomy for suspected ovarian neoplasm. The histopathologic examination of the pertrochanteric nodule confirmed the diagnosis of PMT, while the right ovarian lesion resulted in an AGCT. After surgical excision of both lesions, we observed normalization of serum FGF23 levels and progressive resolution of osteomalacia. It is worth noting that in some cases, after surgery for TIO, as bones are being remineralized the development of a "hungry bone"-like syndrome has been described. This syndrome is characterized by the reduction of serum calcium and magnesium levels, further increase in bALP, decrease of osteoclastic function markers, as well as persistence of osteomalacia symptoms [28]. In our case, it seems that this phenomenon did not occur, as evidenced by the progressive decrease in ALP and bALP levels, along with the relatively rapid improvement of osteomalacia-related symptoms.

In the two reported cases, epithelial ovarian cancer [17] and teratoma [19] were associated with hypophosphatemic osteomalacia and increase in FGF23. In both patients, there was a resolution of hypophosphatemia and normalization of FGF23 levels after tumor excision, similarly to our case. The reduction in FGF23 is consistent with the possibility of using such assay as a marker of disease activity [22]. This is further confirmed by the fact that in a case report by Colangelo et al. [29], using an intraoperative plasma FGF23 assay, a rapid reduction in the levels of this biomarker was observed within one hour from the time of tumor excision, thus suggesting also a possible role of intraoperative FGF23 assay to guide surgery in patients with TIO.

Alongside its function as a marker in TIO, FGF23 has also been hypothesized to play a role in other neoplasms. Since bone is the main site of FGF23 production, malignancies affecting or arising from bone may have a link to FGF23. For example, it has been reported that higher levels of FGF23 in patients with bone metastasis from various solid tumors correlate with decreased survival and shorter time to skeletal-related events [30]. Moreover, osteocyte-derived FGF23 may activate the transcription of pro-metastatic and pro-osteolytic genes in another neoplasm with skeletal involvement, such as multiple myeloma [31].

Regarding ovarian cancers, the FGF/FGF receptor axis has been observed to be a tumor progression mechanism related to angiogenesis, stimulated by secretion of several types of FGF [32]. In addition, elevated serum or plasma FGF23 levels have been documented in women with advanced-stage ovarian cancer when compared to concentrations in women with early-stage ovarian cancer, benign disease, or in healthy women [16]. Immunohistochemistry also detected FGF23 tissue staining in malignant ovarian cancer cells. Interestingly, Tebben et al. [16] report that no patients with ovarian cancer and elevated FGF23 concentrations had hypophosphatemia, suggesting that this increase in FGF23 should be intended in this context more as a marker of disease progression rather than a TIO-related condition.

The literature review thus does not seem to provide conclusive evidence on the association between ovarian cancer, FGF23, and the development of TIO. In our case, immunohistochemistry for FGFR1 on the ovarian neoplasm was not performed, so we have no evidence that this could be a second lesion secreting FGF23 in addition to the PMT in the pertrochanteric region. In any case, the diagnostic investigation for suspected TIO and the presence of unusual uptake in the ovarian area turned out to be a serendipitous occurrence, leading to the early diagnosis and treatment of an AGCT [33].

In conclusion, in patients presenting with clinical features suggestive of adult-onset hypophosphatemic osteomalacia, the diagnosis of TIO should be considered. Functional imaging with PET/CT is useful in identifying a potential PMT, which is the primary cause of TIO. Surgical removal of the PMT, when feasible, is the treatment of choice. However, it is important to consider the possibility that such investigations may identify both FGF23-secreting tumors distinct from PMT and tumors unrelated to the clinical presentation of TIO, which may have a different prognosis and may require dedicated clinical management and treatment.

## Data Availability

The data that has been used is confidential.
